# Disparities in Prevalence of Cardiometablic Risk Factors in Rural, Urban-Poor, and Urban-Middle Class Women in India

**DOI:** 10.1371/journal.pone.0149437

**Published:** 2016-02-16

**Authors:** Indu Mohan, Rajeev Gupta, Anoop Misra, Krishna Kumar Sharma, Aachu Agrawal, Naval K. Vikram, Vinita Sharma, Usha Shrivastava, Ravindra M. Pandey

**Affiliations:** 1 Department of Preventive and Social Medicine, RUHS College of Medical Sciences, Jaipur, Rajasthan, India; 2 Department of Medicine, Fortis Escorts Hospital, Jaipur, Rajasthan, India; 3 Department of Diabetes & Metabolic Diseases, Fortis C-DOC Centre of Excellence, Chiragh Enclave, New Delhi, India; 4 Department of Pharmacology, Rajasthan University of Health Sciences, Jaipur, Rajasthan, India; 5 Department of Home Science, University of Rajasthan, Jaipur, Rajasthan, India; 6 Department of Medicine, All India Institute of Medical Sciences, New Delhi, India; 7 Science and Society Division, Department of Science and Technology, Government of India, New Delhi, India; 8 Department of Biostatistics, All India Institute of Medical Sciences, New Delhi, India; University of Perugia, ITALY

## Abstract

**Objective:**

Urbanization is an important determinant of cardiovascular disease (CVD) risk. To determine location-based differences in CVD risk factors in India we performed studies among women in rural, urban-poor and urban middle-class locations.

**Methods:**

Population-based cross-sectional studies in rural, urban-poor, and urban-middle class women (35–70y) were performed at multiple sites. We evaluated 6853 women (rural 2616, 5 sites; urban-poor 2008, 4 sites; urban middle-class 2229, 11 sites) for socioeconomic, lifestyle, anthropometric and biochemical risk factors. Descriptive statistics are reported.

**Results:**

Mean levels of body mass index (BMI), waist circumference, waist-hip ratio (WHR), systolic BP, fasting glucose and cholesterol in rural, urban-poor and urban-middle class women showed significantly increasing trends (ANOVA_trend_, p <0.001). Age-adjusted prevalence of diabetes and risk factors among rural, urban-poor and urban-middle class women, respectively was, diabetes (2.2, 9.3, 17.7%), overweight BMI ≥25 kg/m^2^ (22.5, 45.6, 57.4%), waist >80 cm (28.3, 63.4, 61.9%), waist >90 cm (8.4, 31.4, 38.2%), waist hip ratio (WHR) >0.8 (60.4, 90.7, 88.5), WHR>0.9 (13.0, 44.3, 56.1%), hypertension (31.6, 48.2, 59.0%) and hypercholesterolemia (13.5, 27.7, 37.4%) (Mantel Haenszel *X*^2^ p_trend_ <0.01). Inverse trend was observed for tobacco use (41.6, 19.6, 9.4%). There was significant association of hypertension, hypercholesterolemia and diabetes with overweight and obesity (adjusted R^2^ 0.89–0.99).

**Conclusions:**

There are significant location based differences in cardiometabolic risk factors in India. The urban-middle class women have the highest risk compared to urban-poor and rural.

## Introduction

Cardiovascular diseases (CVD) are the leading causes of morbidity and premature mortality worldwide [1]. By the year 2025, more than 80% of the global diabetes and CVD burden shall be in low and middle income countries with the bulk being in countries that are undergoing rapid industrialization and urbanization such as India and China [2]. This increase is only partially explained by the increased proportion of older subjects and is mainly due to increasing population levels of risk factors as a result of societal and environmental change [3]. Urban transition (urbanization) is one of the most dramatic shifts in environment that most populations have experienced in the last two centuries. The proportion of individuals living in urban settings has increased markedly worldwide [3]. In the year 1970, the proportion of the world’s population living in urban areas was 37% and is projected to be 61% in 2025. Although urbanization has increased worldwide, the associated societal and health impact is heterogeneous [4]. In high-income countries, urbanization is accompanied by stable economic growth and development of social infrastructure with increased spending on education and healthcare. In low and lower-middle income countries, on the other hand, rapid urbanization has occurred without adequate infrastructure creating urban slums and great socioeconomic disparities. This urban transition is also accompanied by transition in the environment that impacts on behaviors such as diet, physical activity and smoking and leads to an increased prevalence of CVD risk factors [3,5,6].

CVDs are the most important cause of morbidity and mortality in women in all regions of the world, especially high-income countries [1,7]. This is due to high prevalence of multiple cardiovascular risk factors- hypertension, dyslipidemia and diabetes [7]. In India it has been reported that CVDs are the most important cause of death in women, especially in urban regions [8]. It was reported that 16.9% of all deaths in women resulted from cardiovascular disease. The INTERHEART study reported that standard CVD risk factors- smoking, lipid abnormalities, hypertension, diabetes, low physical activity, low fruit and vegetable intake and psychosocial stress–explained more than 90% of incident myocardial infarctions among women [9]. These risk factors are equally important in South Asian patients [10] as well as in women [11]. Regional CVD risk factor epidemiological studies among women in India have reported lower prevalence of diabetes and CVD risk factors as compared to men [12,13]. However, no studies are available that evaluated transition in risk factors in women living in rural, urban slums and urban-middle class locations using similar methodology. Therefore, to study prevalence of multiple CVD risk factors in rural, urban-poor and urban-middle class women using standardized methodology and to evaluate associations of lifestyles with CVD risk factors we performed the present study. This study is secondary analyses of previously published data-sets wherein we evaluated multiple cardiovascular risk factors in rural and urban low socioeconomic status women in the first study [14] and among urban middle-class men and women in the second [15].

## Methods

Two studies were performed using similar methodology. The data regarding prevalence of CVD risk factors for men and women in these studies have been published earlier [14,15]. The present analysis is to determine differences in rural, urban poor and urban middle-class locations. The first study [14] was performed in 5 rural and 4 poor-urban locations in India during the years 2004–2007 and the second [15] a multisite study in urban middle-class locations in 11 cities during the years 2006–10. Details of methodologies of these two studies has been reported earlier [14,15] and important features highlighted below. Ethics committee at All India Institute of Medical Sciences, New Delhi, India and ethics committees at all the study sites approved the first study. Ethics committee at Fortis Escorts Hospital, Jaipur, India, approved the second study. Written consent was obtained from each participant. Sites participating in the study included locations in northern, central, western, eastern and southern regions of India. A proforma was prepared which obtained information regarding social, demographic, diet, physical activity, anthropometric and biochemical variables. This questionnaire has been used in previous epidemiological studies in women in urban slums in Delhi [16] and rural and urban areas of Jaipur [17,18] and has been validated for diet, physical activity, smoking and other assessments. Investigators from each site were centrally trained to ensure uniformity in sampling methodology, questionnaire administration, physical examination and measurements, and biochemical examinations. The study case report form was developed according to recommendations of the World Health Organization [19].

Sampling involved a systematic stratified strategy at each study site. In the first study each site was instructed to identify low and low-middle social status locations in rural and urban areas [14]. The rural locations were in Haryana, Jaipur, Pune, Pondicherry and Gandhigram while urban locations were in Jaipur, Kolkata, Kochi and Pondicherry. For the second study urban middle-class locations were in Jammu, Chandigarh, Bikaner, Jaipur, Ahmadabad, Nagpur, Belgaum, Madurai, Dibrugarh and Lucknow [15]. At the study site a central point was identified and the study investigators moved house-to-house in a clockwise direction from there till the sampling target (n = 500 at each location) was completed. We evaluated middle-aged women 35–70 years at all locations. This strategy has been used in previous studies and has also been recommended by the WHO [18]. The response rate at each site varied from 50 to 70% and was similar in rural and urban locations. In urban area study sample collected by simple cluster sampling using similar methodology.

The questionnaire was designed to collect information on socio-demographic data, history of cardiovascular disease, risk factors and smoking or tobacco intake. Dietary history was inquired using 2-day 24 hour recalls in the first study and questions to inquire dietary fat and fruits and vegetable intake in the second study [14,15]. A set of standardised cups, glasses and spoons was used to assess the intake of each food item. Physical activity was inquired using a previously validated instrument that provides details of all day long activity [20]. This protocol has since been validated for rural women [21] and has been widely used in another large epidemiological study in India [22]. Household chores and work-related physical activity was especially inquired as these can be high in Indian women [21]. Physical examination was performed to assess height, weight, waist and hip size, and blood pressure using techniques recommended by the WHO [23]. All the study investigators were centrally trained in measurement techniques for uniformity. Standardized tape-measures and weighing machines that were periodically calibrated were used. Body mass index (BMI) was calculated as weight (kg) divided by squared height (m). Waist-to-hip ratio (WHR) was calculated. Sitting blood pressure was measured using a calibrated digital sphygmomanometer. Fasting blood sample was obtained in all the study participants. Glucose was determined at the site-based central laboratory using glucose peroxides method and external quality control. Blood levels of and total cholesterol were measured using cholesterol oxidase-phenol 4-aminophenazone peroxidase methods respectively with quality control as reported earlier [14,15].

Smokers included subjects with present smoking and regular non-smoked tobacco use. Former tobacco users were also identified. Physical activity was classified as moderate or severe if the woman was involved in moderate intensity household work of walking >30 minutes/day [22]. In women, overweight was defined as body mass index (BMI) ≥25 kg/m^2^ and obesity defined as BMI ≥30 kg/m^2^[23]. Abdominal obesity was defined using two definitions: waist: hip ratio (WHR) of >0.8 for mild and >0.9 was moderate or waist circumference >80 cm for mild or >90 cm for moderate [23]. Hypertension was diagnosed when the systolic or diastolic BP was ≥ 140 and/or ≥90 mmHg on multiple single day measurements or the subject was known hypertensive on medications [24]. Dyslipidemia was defined by the presence of high total cholesterol (≥200 mg/dl, 5.2 mmol/L) according to the National Cholesterol Education Program guidelines [25]. Diabetes was diagnosed in women with previously diagnosed diabetes or fasting blood glucose ≥ 126 mg/dl or >7.0 mmol/L [25].

### Statistical analyses

All the case report form data were entered in a customized database using SPSS program (SPSS Inc. Chicago, USA). All the analyses have been performed using SPSS Version 10.0. Age adjusted mean levels of different demographic, lifestyle and physical variable were determined. Numerical variables are reported as mean ±SEM. Trends in numerical variables have been determined by ANOVA test for trend. Prevalence rates are reported as percent with 95% confidence intervals (CI). Age-adjustment in prevalence rates has been performed using the direct method and female population in 2001 Indian census used for adjustment. We used these age-data as it corresponds better with the study periods. Trends in prevalence of risk factors in rural, urban-poor and urban-middle class groups were determined with Mantel Haenszel *X*^*2*^ test for trend. Two-line regression analysis was performed to determine association of overweight/obesity in rural, urban poor and urban middle class women with various risk factors. P values <0.05 were considered significant.

## Results

We evaluated 6853 women (rural 2616, urban poor 2008 and urban middle class 2229) at the various study sites. Response rates and details of recruitment at different sites has been reported [14,15]. At rural, urban poor and urban middle-class locations, respectively, women in age groups 35–49 were 55.1, 58.8 and 43.4%, in age groups 50–59 were 23.3, 23.8 and 25.7% and in age group ≥60 were 15.7, 17.3 and 24.5%. Illiteracy and low educational status (<10 yr formal education) was significantly greater in rural women (96.9%) compared to urban poor (83.7%) and the urban middle class (47.6%) (p<0.05).

Table 1 shows the age adjusted mean±SEM values and 95% confidence intervals of various anthropometric and physical and biochemical variables in all the 3 groups. All the physical variables (weight, height, waist size, hip size, BMI and WHR are significantly greater in the urban middle-class women as compared to urban poor and the rural (p<0.01). There is evidence of significantly increasing trends in BMI and WHR from rural to urban poor and urban middle-class groups (ANOVA test for trend, p<0.001). Significantly increasing trends are also observed for mean levels of systolic BP, diastolic BP, fasting glucose and total cholesterol levels (ANOVA test for trend, p<0.001, Table 1).

**Table 1:** Age adjusted values (mean±SEM, 95% confidence intervals) of anthropometric and biochemical variables in rural, semi-urban and urban women.

Variable	Rural (n = 2616)	Urban poor (n = 2008)	Urban middle class (n = 2229)	ANOVA F value	P value for trend
**Anthropometry**					
Height (cm)	150.8±0.12 (150.5–151.0)	151.5±0.13 (151.3–151.8)	153.8±0.18 (153.6–153.9)	119.3	<0.0001
Weight (kg)	50.6±0.22 (50.1–51.30)	56.6±0.25 (56.0–57.0)	62.4±0.26 (62.1–62.6)	627.0	<0.0001
Waist (cm)	74.5±0.23 (74.1–75.0)	83.4±0.26 (82.9–83.9)	86.1±0.23 (85.9–86.3)	678.6	<0.0001
Hip (cm)	90.4±0.21 (90.0–90.8)	94.5±0.23 (94.1–95.0)	94.4±0.3 (94.1–94.6)	105.3	<0.0001
Body mass index (kg/m^2^)	22.2±0.1 (22.0–22.4)	24.6±0.1 (24.4–24.8)	26.6±0.1 (26.4–26.7)	435.9	<0.0001
Waist-hip ratio	0.83±0.01 (0.82–0.83)	0.88±0.01 (0.88–0.88)	0.92±0.01 (0.91–0.92)	31.1	<0.0001
**Physical and biochemical factors**					
Systolic blood pressure (mmHg)	124.0±0.4 (123.3–124.7)	126.7±0.4 (125.9–127.5)	129.2±0.3 (128.9–129.6)	49.6	<0.0001
Diastolic blood pressure (mmHg)	79.5±0.2 (79.1–79.9)	81.5±0.3 (81.1–82.0)	81.9±0.2 (81.68–82.06)	39.3	<0.0001
Glucose fasting (mg/dl)	87.1±0.5 (86.0–88.1)	97.2± 0.6 (96.0–98.3)	108.6±1.0 (107.6–109.6)	251.4	<0.0001
Cholesterol (mg/dl)	166.5±0.6 (165.2–167.9)	180.7±0.7 (179.3–182.2)	189.6±0.82 (188.7–190.4)	260.6	<0.0001

Age-adjusted prevalence of various risk factors and diabetes in rural, urban poor and urban middle-class women is shown in Table 2. In rural, urban-poor and urban-middle class women, respectively, prevalence of various risk factors is overweight/obesity (BMI ≥25 kg/m^2^) in 22.5, 45.6 and 57.4%, waist circumference >80 cm in 28.3, 63.4 and 61.9%, waist circumference >90 cm in 8.4, 31.4 and 38.2%, WHR >0.8 in 60.4, 90.7 and 88.5%, WHR >0.9 in 13.0, 44.3 and 56.1%, hypertension in 31.6, 48.2 and 59.0%, hypercholesterolemia in 13.5, 27.7 and 37.4% and diabetes in 2.2, 9.3 and 17.7%. We also performed correlation of prevalence rates of various cardiovascular risk factors with degree or urbanization using Mantel Haenszel X^2^ for trend. Significant increasing trend in prevalence of these cardiometabolic risk factors with increasing urbanization is observed (X^2^ for trend p<0.01 for all). Any tobacco use (smoking or smokeless tobacco) shows a declining trend with urbanization- 41.6, 19.6 and 9.4% (p<0.01).

**Table 2:** Age adjusted prevalence (%, 95% confidence intervals) of risk factors in rural, poor-urban and urban women.

Variable	Rural (n = 2616)	Poor-urban (n = 2008)	Urban (n = 2229)	χ^2^ (P value)
**Lifestyle factors**				
Current tobacco users	41.6(39.5–43.7)	19.6(17.8–21.6)	9.4(8.3–10.7)	294.0(<0.001)
Smoking	10.6(9.5–11.8)	0.7(0.4–1.2)	0.6(0.35–1.0)	337.0(<0.001)
Non-smoked tobacco use	23.2(21.6–24.9)	16.2(14.6–17.9)	8.8(7.7–10.0)	131.2(<0.001)
Former tobacco users	2.1(1.5–2.8)	1.1(0.7–1.7)	0.9(0.6–1.4)	227.3(<0.001)
Sedentary lifestyle	60.1(58.2–62.0)	71.0(69.0–73.0)	41.40(39.4–43.5)	170.3(<0.001)
**Anthropometry**				
BMI 25.0–29.99 kg/m^2^	16.8(15.4–18.3)	31.7(29.7–33.8)	37.7(35.7–39.8)	147.7(<0.001)
BMI ≥30.0 kg/m^2^	5.7(4.9–6.6)	13.9(12.5–15.5)	19.7(18.1–21.4)	161.51(<0.001)
Waist:hip ratio >0.8	60.4(58.5–62.3)	90.7(89.3–91.9)	88.5(87.1–89.8)	112.0(<0.001)
Waist:hip ratio >0.9	13.0(11.7–14.4)	44.3(42.1–46.5)	56.1(54.0–58.1)	554.0(<0.001)
Waist size > 80 cm	28.3(26.6–30.1)	63.4(61.2–65.4)	61.9(59.8–63.9)	277.3(<0.001)
Waist size > 90 cm	8.4(7.4–9.5)	31.4(29.4–33.4)	38.2(36.2–40.3)	384.9(<0.001)
**Risk factors**				
Hypertension	31.6(29.7–33.5)	48.2(46.0–50.4)	59.0(57.0–61.0)	158.3(<0.001)
Cholesterol ≥200 mg/dl	13.5(12.2–14.9)	27.7(25.8–29.7)	37.4(35.4–39.4)	247.2(<0.001)
Diabetes	2.2(1.7–2.9)	9.3(8.1–10.7)	17.7(16.1–19.4)	241.1(<0.001)

There is a significant correlation (two-line regression) of greater overweight and/or obesity in urban poor and urban middle class women with prevalence of hypertension (adjusted R^2^ = 0.99, p = 0.040), hypercholesterolemia (R^2^ = 0.98, p = 0.049) and diabetes (R^2^ = 0.89, p = 0.048). A weaker correlation is observed with greater prevalence of abdominal obesity (high waist circumference) with hypertension (R^2^ = 0.93, p = 0.116), hypercholesterolemia (R^2^ = 0.92, p = 0.125) and diabetes (R^2^ = 0.76, p = 0.124).

**Fig 1:**
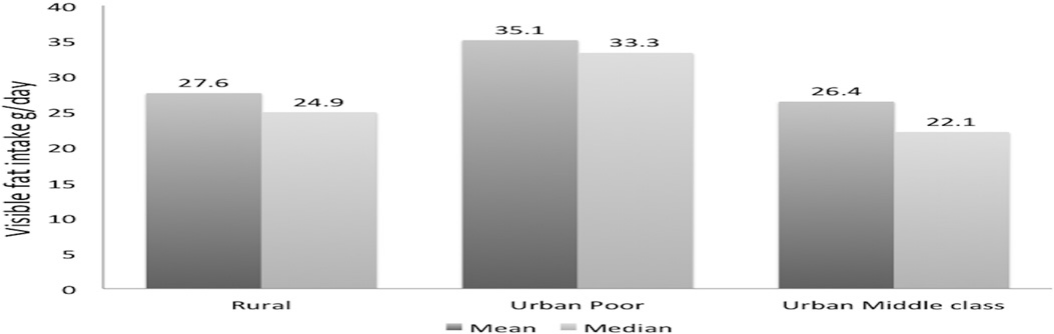
Dietary visible fat intake in the rural, poor urban and urban women at Jaipur study site (mean and median intake, g/day).

We also determined prevalence of physical inactivity and unhealthy diet in various groups (Table 2). Urban poor women had significantly greater prevalence of physical inactivity and unhealthy diet as compared to rural women (p<0.05). Similar data from the urban middle-class women were not available and among these women a high prevalence of physical inactivity as well as high fat and low fruit/vegetable diet was reported using different criteria [15]. Among urban middle class women low to mild physical activity was in 36.4% and 47.0% while 16.6% were moderately active. Dietary intake of visible fat of 20–40g/day was in 34.7% and >40g/day in 13.5% [15]. In a sub-sample at Jaipur site we evaluated intake of visible fats in rural, urban poor and urban middle-class women (Fig 1). The median visible fat intake in rural, urban-poor and urban middle-class women was 24.9, 33.3 and 22.1 g/day, while mean intake was 27.6±11.6, 35.1±14.9 and 26.4±14.4 g/day, respectively (p<0.01 for rural and urban women vs. urban-poor).

## Discussion

Worldwide, women are known to have high prevalence of diabetes, cardiovascular disease and its metabolic risk factors [5]. In India, although mortality data have reported importance of cardiovascular disease in women [8], it is only now being realized that these conditions are the commonest cause of premature mortality in women as reported in the latest iteration of Global Burden of Diseases Study [26]. The present study shows high prevalence of multiple cardiovascular risk factors, including diabetes, in urban middle-aged women in India. The prevalence of obesity, abdominal obesity, hypertension, hypercholesterolemia and impaired fasting glucose is significantly greater in urban middle-class and urban-poor women compared to the rural. There is a significantly increasing trend in all these metabolic factors with increasing urbanization. This corresponds with increasing levels of generalized and abdominal obesity, which are surrogates for greater dietary calorie and fat consumption and lower physical activity.

This is one of the first studies that systematically evaluated cardiovascular risk factors among women in all regions of India. The results show that urban women have the highest prevalence of risk factors and there is a clear rural to urban-poor to urban-middle class gradient in risk factors (Table 1). The urbanization gradient in risk factors that we have observed is a unique feature in South Asian men and women and reflects large socioeconomic disparities among these three groups [27–29]. This is also one of the first studies that has evaluated lifestyle determinants of urban-rural differences in India using similar methodologies and has identified that lifestyle factors are important. Only a few small studies have systematically studied urban-rural differences in India and most did not study comprehensive risk factor profile especially amongst women [12]. This is also one of the first studies that evaluated middle aged women, a segment of the society that has been neglected in previous studies limited to younger or older age groups [13]. In India, more than 90% of populations live in areas similar to the study subjects and therefore the data are representative of the majority of women in India.

Urbanization is one of the most dramatic demographic changes occurring in developing countries such as India [30]. Greater prevalence of diabetes and other CVD risk factors with increasing urbanization is due to multiple factors [3,5]. Changes in diet have been attributed to economic growth leading to changes in food consumption, relative cost, availability and media and industry influences [31]. Changes in physical activity have been attributed to mechanization at work and home [5]. Change in transportation (e.g. increased motorised vehicle ownership), and changes in the built environment (e.g. increased urban sprawl and poor connectivity in residential areas) also lead to lower physical activity [31]. Greater tobacco use in rural women is reflection of lower literacy and is also influenced by environmental factors such as tobacco policy and greater social acceptability of smoking [32]. There are limited studies that have similarly evaluated role of urbanization and transition in risk factors in women. Increasing urbanicity within an urban population has been reported to be associated with greater prevalence of diabetes in South India and Sri Lanka [33,34]. Greater urban social development index as well as human development index has also been reported to be associated with greater cardiometabolic risk factors in India [35]. On the other hand, although multiple studies have evaluated urban-rural differences in risk factors in men and women in India, they are smaller than the present study [13]. Moreover, the present study has evaluated women at different sites of the country and is more representative.

In developed countries, the highest prevalence of CVD risk factors is observed amongst the less literate and low socioeconomic subjects living in downtown urban locations [5,6]. We observed that less literate and poorer rural women had lower prevalence of risk factors. This follows the observation that the transition from low cardiovascular risk to high-risk in low socioeconomic subjects tracks the gross national income and only when the gross national income of a country exceeds a certain amount (e.g., 10,000 international dollars per annum) does the inversion of risk factors take place [36]. Increased incomes lead to unhealthy diet as well as decline in physical activity. In India, increased intake of fats, saturated fats and calories and decline in physical activity with greater urbanization has been reported [37]. India has one of the highest social and income inequities in the world [27] and it is likely that rural-urban differences in CVD risk factors are due to this reason. Marmot attributes the difference in chronic disease risk factors in different social classes to a variety of factors such as hierarchy, isolation, instability, low social support and poor cohesion [38]. In India, the rural societies are still socialist, stable, supportive and cohesive and it is likely that lower prevalence of risk factors is their reflection [39]. We did not study these risk factors and cannot comment on them. Other factors that explain urban-rural differences in low income countries are community and environmental influences (pollution policies, tobacco policies, food policies, social and cultural norms, built environment, safety, land use, food choices, unhealthy food supply, portion sizes, salt consumption, etc.), social factors (networks, access to healthcare, poverty, etc.) and individual behavior related to smoking, diet, psychological stress and healthcare seeking and compliance with therapies [40]. We did not study these multiple determinants and cannot comment on their importance in the present study. Other limitations of the study are related to sampling bias, small sample sizes at individual location and variable response rates. These limitations have been discussed earlier [14,15]. External validity of our findings can be questioned as our study did not include poor Indian states such as Orissa, Bihar and Uttar Pradesh and this is a study limitation. On the other hand, these are the largest data-set on cardiovascular risk factors among women in India and includes many large Indian states.

In conclusion, this is one of the largest studies of prevalence of multiple cardiovascular risk factors in Indian women and shows that there is an urbanization related transition of overweight/obesity, abdominal obesity, hypertension, hypercholesterolemia, impaired fasting glucose and diabetes. This transition is associated with greater fat consumption and lower physical activity. Control of unhealthy consequences of this transition shall require innovative strategies that promote healthy urbanization with focus on macrolevel as well as microlevel environments, that promote physical activity and improve availability and intake of healthy foods.
